# Genetic Identification Is Critical for the Diagnosis of Parkinsonism: A Chinese Pedigree with Early Onset of Parkinsonism

**DOI:** 10.1371/journal.pone.0136245

**Published:** 2015-08-21

**Authors:** Yang Yang, Bei-sha Tang, Ling Weng, Nan Li, Lu Shen, Jian Wang, Chuan-tao Zuo, Xin-xiang Yan, Kun Xia, Ji-feng Guo

**Affiliations:** 1 Department of Neurology, Xiangya Hospital, Central South University, Changsha, 410008, Hunan, People’s Republic of China; 2 State Key Laboratory of Medical Genetics, Changsha, 410078, Hunan, People’s Republic of China; 3 Key Laboratory of Hunan Province in Neurodegenerative Disorders, Central South University, Changsha, 410008, Hunan, People’s Republic of China; 4 Neurodegenerative Disorders Research Center, Central South University, Changsha, 410008, Hunan, People’s Republic of China; 5 Department of Neurology, Huashan Hospital, Fudan University, 12 Wulumuqi Middle Road, 200040, Shanghai, People’s Republic of China; 6 PET Center, Huashan Hospital, Fudan University, 12 Wulumuqi Middle Road, 200040, Shanghai, People’s Republic of China; Emory University, UNITED STATES

## Abstract

**Background:**

A number of hereditary neurological diseases display indistinguishable features at the early disease stage. Parkinsonian symptoms can be found in numerous diseases, making it difficult to get a definitive early diagnosis of primary causes for patients with onset of parkinsonism. The accurate and early diagnosis of the causes of parkinsonian patients is important for effective treatments of these patients.

**Methods:**

We have identified a Chinese family (82 family members over four generations with 21 affected individuals) that manifested the characterized symptoms of parkinsonism and was initially diagnosed as Parkinson’s disease. We followed up with the family for two years, during which we carried out clinical observations, Positron Emission Tomography-Computed Tomography neuroimaging analysis, and exome sequencing to correctly diagnose the case.

**Results:**

During the two-year follow-up period, we performed comprehensive medical history collection, physical examination, and structural and functional neuroimaging studies of this Chinese family. We found that the patient exhibited progressive deteriorated parkinsonism with Parkinson disease-like neuropathology and also had a good response to the initial levodopa treatment. However, exome sequencing identified a missense mutation, N279K, in exon 10 of *MAPT* gene, verifying that the early parkinsonian symptoms in this family are caused by the genetic mutation for hereditary frontotemporal lobar dementia.

**Conclusions:**

For the inherited parkinsonian patients who even show the neuropathology similar to that in Parkinson’s disease and have initial response to levodopa treatment, genetic identification of the molecular basis for the disease is still required for defining the early diagnosis and correct treatment.

## Introduction

Parkinsonism is a neurological syndrome characterized by tremor, hypokinesia, rigidity, and postural instability. In addition to drug- or toxin-induced parkinsonism, a wide range of diseases may lead to a similar set of symptoms, including Parkinson’s disease, parkinsonism-plus, Wilson’s disease, progressive supranuclear palsy, and a handful of other neurological conditions [1]. The fact that parkinsonism could be the only symptom in the early stage of numerous diseases makes it difficult to get a definitive early diagnosis for the primary causes of parkinsonism. However, as the disease progresses, some other significant characteristics of the symptoms would come forth; therefore, many diagnoses rely on the disease progression and close clinical observations for identifying the underlying causes of patients with parkinsonism. Although the fast development of functional neuroimaging technologies, including MRI, Positron Emission Tomography-Computed Tomograph (PET-CT) and single-photon emission CT (SPECT) provide us with high precision approaches that are required in modern clinical practice, the early diagnosis of Parkinsonian patients can be achieved by genetic identification of the gene mutations. Mutations in genes at more than 20 loci are known to cause genetic parkinsonism [2,3], however, the number of corresponding genes that need to be screened could be substantially high. Compared to the traditional candidate gene screening that is expensive and ineffective, the next generation sequencing methods, such as whole-genome sequencing and exome sequencing, should allow for readily identifying the genetic bases of Mendelian diseases.

Here we report the use of continued clinical observations, especially the functional neuroimaging studies, and the application of next generation sequencing methods to identify the genetic cause of a family with early parkinsonian symptoms.

## Materials and Methods

### 2.1 Subjects and Clinical Text

The study was approved by the Ethics Committee of Xiangya Hospital affiliated to Central South University in China. Written informed consents were obtained from all subjects.

**Fig 1:**
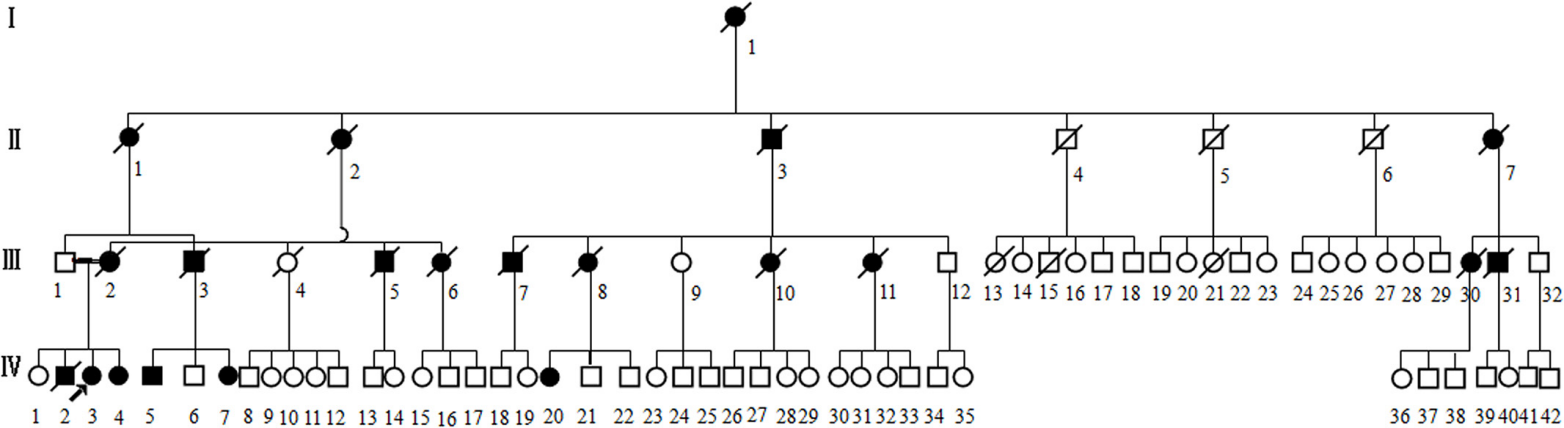
Pedigree of the family. Squares represent male family members; circles, female family members; filled symbols, affected subjects; a diagonal line through a symbol, deceased; short arrow, proband.

We collected a four-generation Chinese family (Fig 1) with parkinsonism, characterized by early onset, rapid progression, rigidity, hypokinesia, postural instability, and in some individuals, tremor. All the available affected individuals (5 patients, including 1 male and 4 female) were subjected to thorough neurological examinations by two or more experienced neurologists. Data from other family members were collected via interviews and available medical records of deceased patients. Genomic DNA was obtained from two groups of affected and unaffected individuals.

### 2.2 Neuroimaging

Magnetic Resonance Imaging (1.5T MRI scanner Signa GE, Milwaukee, USA) was performed in Ⅳ3 (proband) and Ⅳ20, and PET-CT imaging was carried out in Ⅳ1, Ⅳ3 and Ⅳ20. In MRI examination, the T1-weighted (T1W), T2-weighted (T2W) studies were available in our cases. ^11^C-CFT uptake studies and PD-related covariance pattern (PDRP) based on ^18^F-fluorodeoxyglucose (FDG) positron emission tomography (PET) scans from Ⅳ1, Ⅳ3 and Ⅳ20 were performed in three-dimensional (3D) mode on a Siemens Biograph TruePoint PET•CT (Siemens healthcare, Munich, Bavaria, Germany), producing image slices over the whole brain with an intrinsic 3D resolution.

### 2.3 Exome Sequencing

Qualified genomic DNA samples were extracted from peripheral blood leucocytes of two affected members. The library preparation and exome sequencing, alignment of sequenced reads, single-nucleotide polymorphism and insertions or deletions calling, analysis for exome-sequencing results were processed as previously described [4].

### 2.4 Segregation Analysis

Seven variants predicted to be damaging, including exon 10 of the *MAPT* gene, were amplified by means of a polymerase-chain-reaction (PCR) assay and directly sequenced in all members of the study family.

## Results

### 3.1 Clinical Presentations

The pedigree consisted of 82 family members over four generations, with 21 affected individuals, among which 5 patients were available (Fig 1). Static tremor on left lower limb was the initial symptom found in the right-handed proband (Ⅳ3) at age 39. She suffered from progressive bradykinesia and rigidity over trunk and left limb one year later, described as deteriorated ability of balance which led to occasional falls. Cramp in left lower limb started at the early stage of the disease. The diagnosis of “Parkinson’s disease” was made at the age of 41, and she was administered with levodopa in combination with benserazide, piribedil, and benzhexol hydrochloride to which she responded well initially. When she was first admitted to our institute in 2008, at 42 years old, she presented with unilateral resting tremor, mild bradykinesia, rigidity exhibited in axial muscles, and postural instability, spared from speech or eye movement involvement. The UPDRS motor part was 10 points and MMSE score was 30 of 30 points (Table 1).

**Table 1:** Clinical features in five affected members of the family.

	Ⅳ3	Ⅳ4	Ⅳ5	Ⅳ7	Ⅳ20
Sex	F	F	M	F	F
Age at exam (yr)	44	42	44	42	47
Age at onset (yr)	39	41	42	40	45
Disease duration(yrs)	5	1.5	2	2	2
Initial symptoms	tremor	tremor	rigidity	rigidity	bradyinesia
Parkinsonism					
Bradykinesia	+	+	+	±	++
Rigidity	+	±	++	++	++
Tremor	+	+	+	-	+
Postural instability	++	+	++	+	+
Response to dopaminergic therapy	++	++	-	-	+
Personality changes	++	++	+	+	++
Dementia	++	+	ND	-	++
Eye movement abnormalities	++	+	++	-	-
Blurred vision	++	+	++	+	-
Nystagmus	+	-	-	-	-
Dysarthria	+	±	++	±	++
Mutism	++	-	+	-	++
Drooling	+	±	+	-	+
Dystonia	+	+	-	-	-
Seizures	-	-	-	-	-
Ankle edema	-	-	-	-	+++
Urinary incontinence	-	-	-	-	+
Tendon reflexes	N	↑lower limbs	↑upper limbs	↑upper limbs	N
Babinski’s sign	+	±	-	±	+
Frontal lobe release sign	+	+	+	+	+
Cerebellar signs	-	-	-	-	-
UPDRS motor (part Ⅲ)	16	23	ND	13	54
Webster Score	7	10	ND	4	21
MMSE	29/30	30/30	ND	30/30	16/30
WAIS-RC	Inferior to the bottom 5% of the population	Inferior to the bottom 20% of the population	ND	ND	ND

Notes: Clinical signs are graded as follows: − = absent, ± = subtle, + = mild, ++ = moderate, +++ = severe, ↑ = increased, N = normal, D = defect, ND = not determined, UPDRS = Unified Parkinson’s Disease Rating Scale, WAIS-RC = Chinese Wechsler Intelligence Scale.

The proband was re-examined at 44 years old, in 2010. Her symptoms were deteriorated. Static tremor was still present, but had affected both lower extremities. Stiffness in the muscles of her neck, tongue and lower limb was conspicuous. Blurred vision was first demonstrated at the age of 43, accompanied by progressive visual acuity deterioration. Though described by her family as social withdrawal and apathy, she was still independent in most of her daily activities. During examination, occasional drooling, paucity of facial expression and severely reduced blinking rate were observed. Absent vertical gaze and slow horizontal gaze were displayed, with discontinued horizontal nystagmus. Mutism was notable, as well as hypophonic voice. Muscle tone was increased, while muscle strength remained normal. Increased tendon reflexes were obvious whereas Babinski sign was equivocal bilaterally. Frontal lobe release signs were present, including glabellar and palmomental reflexes. UPDRS motor part was 16 points. She scored 29 of 30 points on the MMSE test, missing one point on the recall scales. However, the further Chinese Wechsler Intelligence Scale (WAIS-RC) showed that she scored in the bottom fifth percentile of the population.

The clinical features of the five available affected family members are summarized in Table 1. Adding data from the historical records of deceased patients, the age of onset of all patients in the family ranged from 39–48 years (mean 43.7 ± 2.9 years) and disease duration before death varied from 4–11 years (mean 7 ± 0.6 years). Parkinsonian features were illustrated as one of the most predominant initial symptoms in almost all patients. After onset, all affected individuals displayed a rapid progression. Personality changes, which included apathy, irritability, excessive adherence, disinhibition, were prominent in the early or middle stage, and worsened as disease developed. All patients showed postural instability and some were reported to exhibit positive Babinski sign. Supranuclear gaze palsy was presented in some affected individuals in the later stage. No record of cognitive functions of deceased patients was available, but some of these patients were recalled as having diminished memory by their families. WAIS-RC and MMSE of the proband and two other patients showed intellectual deficits.

### 3.2 Structural and Functional Neuroimaging Studies

**Fig 2:**
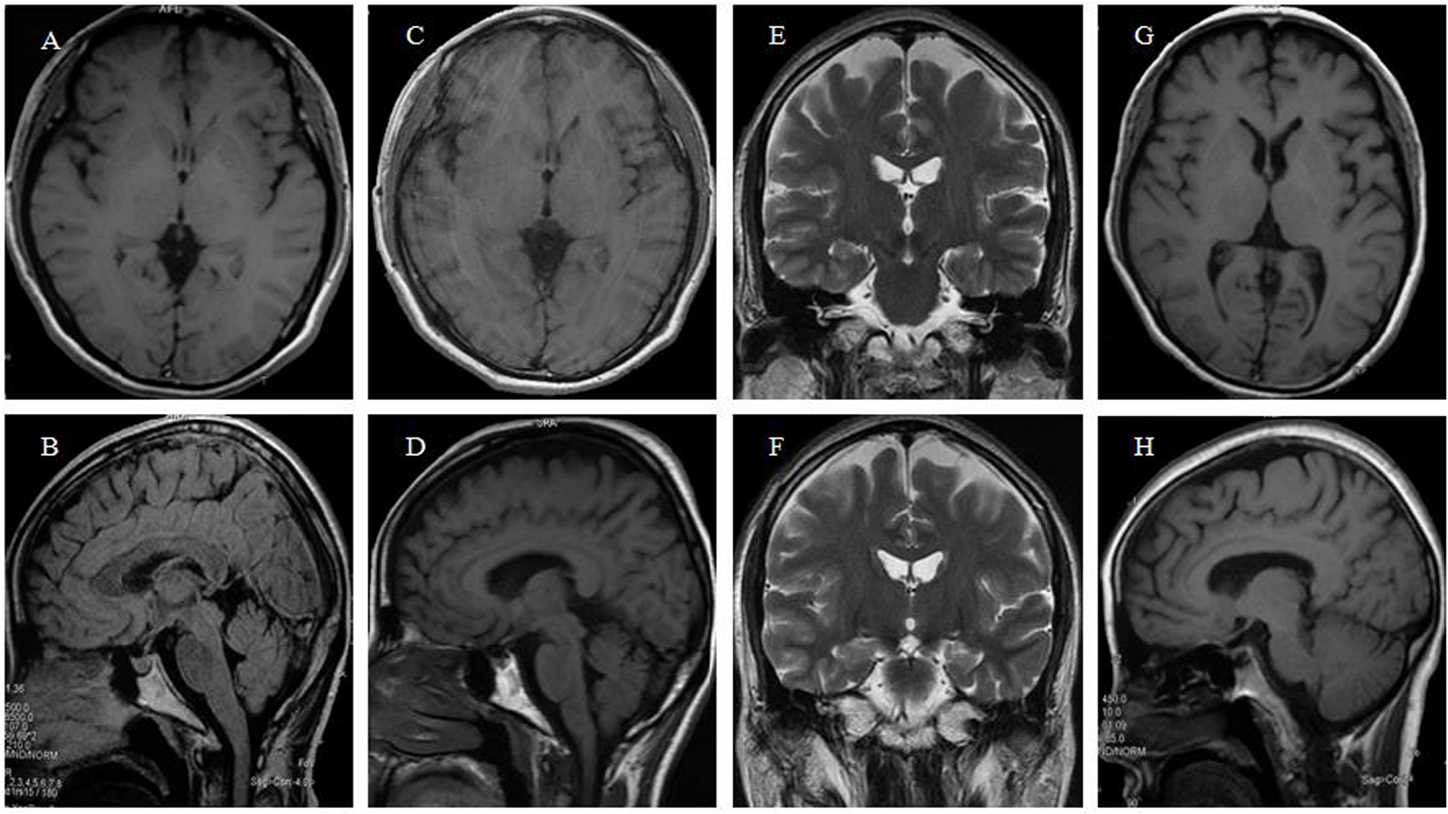
Brain MRI and CT scans of two patients of the family. A,B: brain MRI scan of the proband at the age of 42; C-F: brain MRI scan of the proband at the age of 44; G,H: brain MRI scan of the patient Ⅳ20 at the age of 46. Atrophy of whole brain was present in both patients. The frontotemporal atrophy was the most noticeable, while in the proband the hippocampus was relatively well-preserved.

**Fig 3:**
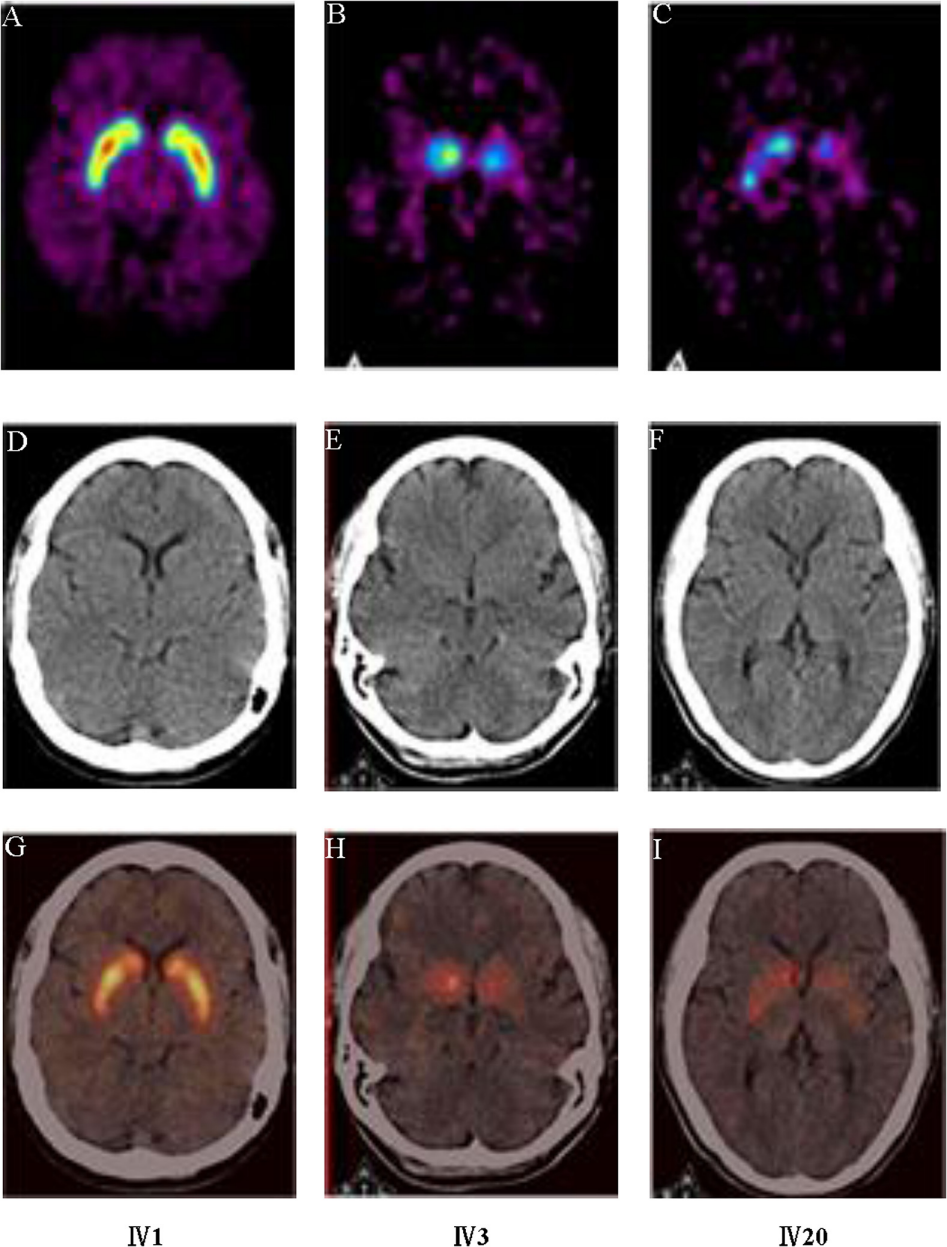
PET imaging of DATs via ^11^C-CFT in pedigree with p.N279K *MAPT* mutation. A-C: Axial positron emission tomographic images of ^11^C-CFT uptake in three family members, A, Ⅳ-1, B, Ⅳ-3, C, Ⅳ-20; D-F: The transverse CT in three family members, D, Ⅳ-1, E, Ⅳ-3, F, Ⅳ-20; G-I: Integrated imaging of Axial positron emission tomographic images and transverse CT in three family members, G, Ⅳ-1, H, Ⅳ-3, I, Ⅳ-20. The region with the highest signal intensity indicates the intake of ^11^C-CFT by regional dopamine transporter.

**Fig 4:**
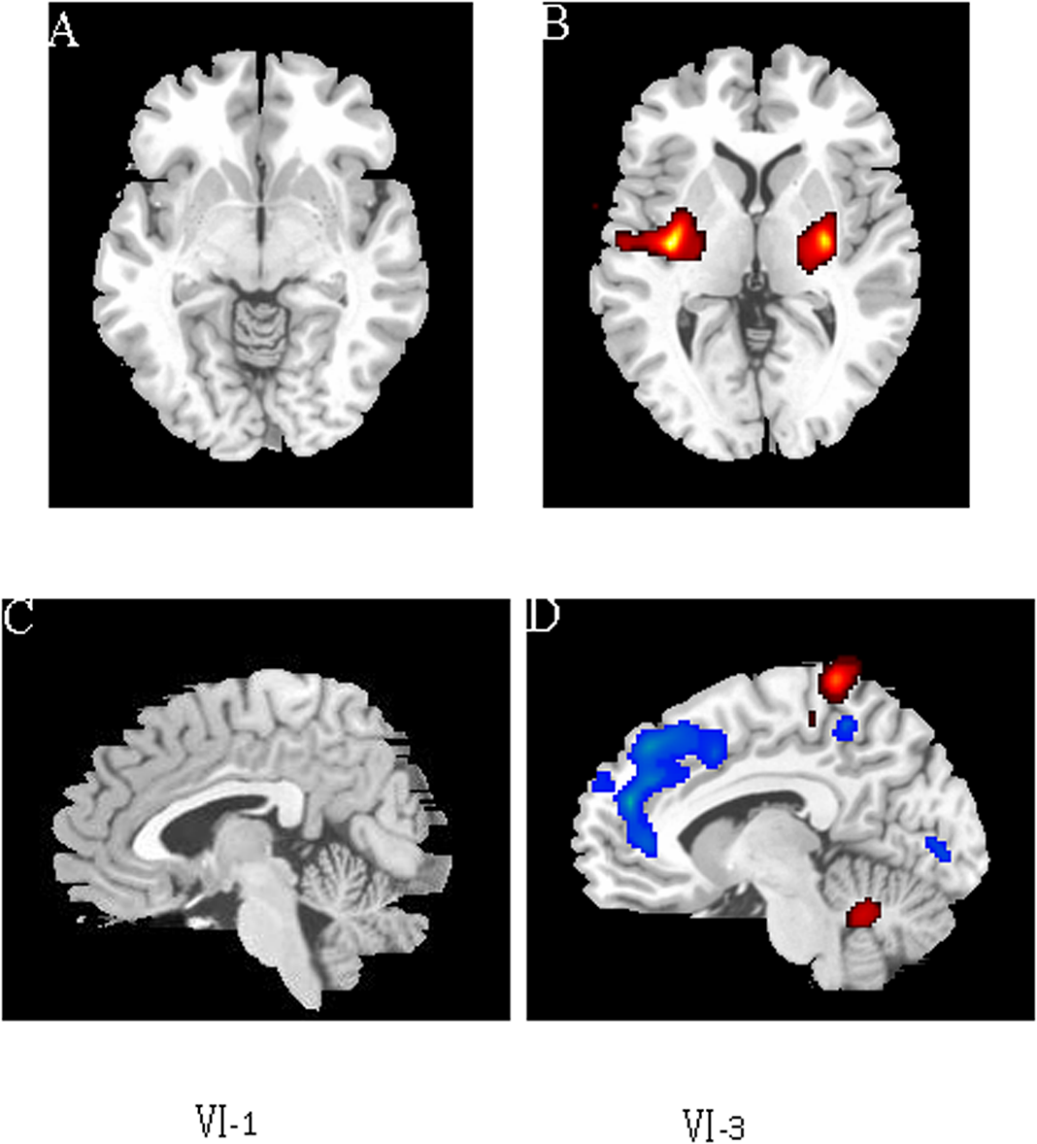
PDRP via ^18^F-FDG in pedigree with p.N279K *MAPT* mutation. Parkinson’s disease-related pattern (PDRP) identified by network analysis of ^18^F-FDG PET scans from two family members–patient Ⅳ-3 matched with non-patient Ⅳ-1. A, B: Axial PDRP of [^18^F]-FDG PET scans from two family members, A, Ⅳ-1, B, Ⅳ-3; C, D: Sagittal PDRP of [^18^F]-FDG PET scans from two family members, C, Ⅳ-1, D, Ⅳ-3. Red signals in putamen (B), globus pallidum (B), cerebellum (D) and sensorimotor cortex (D) indicate the brain regions with increased metabolic activity, whereas blue signals in extensive prefrontal areas (D) indicate the brain regions with decreased metabolic activity.

The MRI scan of the proband at 42 years old demonstrated atrophy of the whole brain. It’s worth pointing out that the atrophy of frontotemporal lobe is most noticeable while the hippocampus is relatively unaffected (Fig 2A and 2B). In her second MRI scan, the results revealed the correspondent deterioration of patient’s condition, exhibiting the aggravated atrophy of frontotemporal lobe (Fig 2C–2F). Furthermore, we obtained the MRI image data from another family member Ⅳ20, who had very similar pattern of brain atrophy as well (Fig 2G and 2H). We also performed functional neuroimaging studies in the two patients. We performed the PET images via ^11^C-CFT in the pedigree, including one unaffected member Ⅳ1 and two patients Ⅳ3 and Ⅳ20 (Fig 3), and the results of the quantitative ^11^C-CFT uptake demonstrated that Ⅳ3 (Fig 3H) and Ⅳ20 (Fig 3I) exhibited significantly decreased uptake ratio of ^11^C-CFT in the striatum (specifically the caudate nucleus and putamen) compared with that of Ⅳ1 (Fig 3G), which was within the normal range (Table 2), indicating that the DAT availability of affected individuals was reduced in the nigrostriatal projection area. Furthermore, PDRP analysis via ^18^F-FDG PET (Fig 4) showed that in Ⅳ3, significant hypometabolism was identified in extensive prefrontal areas (Fig 4D), whereas hypermetabolism was found in the putamen, globus pallidum, cerebellum and sensorimotor cortex (Fig 4B and 4D) as compared with the normal member Ⅳ1 (Fig 4A and 4C). The overactivated regions were in accordance with the PDRP patterns found in Parkinson’s disease patients [5–7].

**Table 2:** Quantitative comparison of ^11^C-CFT uptake in the family members.

Subject	Ratio					
	Caudate nucleus		Putamen(anterior)		Putamen(Posterior)	
	Left	Right	Left	Right	Left	Right
Ⅳ-1	3.0	3.24	3.71	3.86	3.43	3.33
Ⅳ-3	1.63	1.78	1.11	1.22	1.04	1.11
Ⅳ-20	1.57	1.76	1.43	1.38	1.38	1.71
Range of normal value	2.51–3.41		3.22–4.26		3.11–4.05	

### 3.3 Genetic Variations Identified by Exome Sequencing

**Fig 5:**
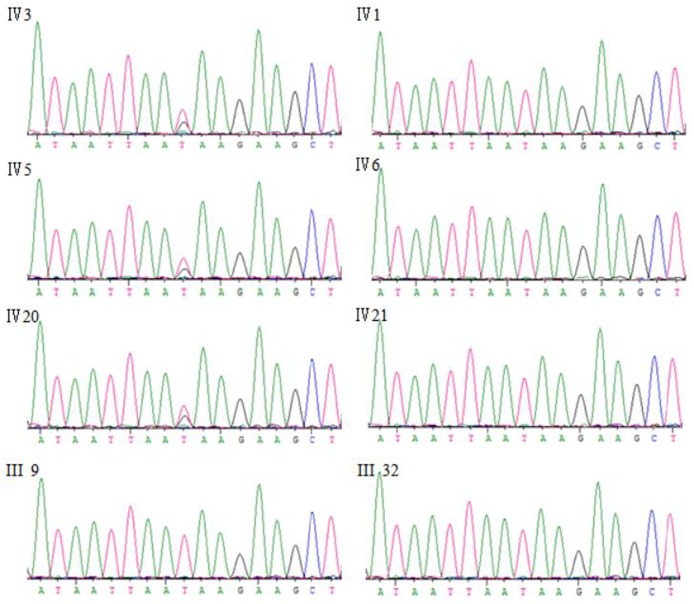
Sanger sequences of *MAPT* p.N279K mutation in this family. Ⅳ-3, Ⅳ-5, Ⅳ-20 are patients carrying the *MAPT* p.N279K mutation; IV-1, IV-6, IV-21, III-9, III-32 are unaffected family members.

Exome sequencing was performed for the two affected individuals in the family (Ⅳ3, Ⅳ20 in Table 3). The quality of variations was controlled by the criteria previously established [4]. Using *de novo* assembly of exon sequences, we detected 114 coding insertions and 198 deletions. After identification of variants, we focused only on NS/SS/Indel (non-synonymous/donor site mutations and splicing/frameshift) variants which were more likely to be pathogenic mutations than other variants. Each sample was found to have at least a single NS/SS/Indel variant in ~6,900 candidate genes. Given that this is a rare disorder it is unlikely that the causative variants will be present in the general population, we compared our NS/SS/Indel variants to dbSNP129, eight previously exome-sequenced HapMap samples (‘HapMap 8’), and the SNP release of the 1000 Genome Project (20100208 release) (Table 3), and removed the shared SNPs. Because parkinsonism in this family is autosomal dominantly inherited, all affected individuals should share the same causal variant, thus comparison of exome data from two samples (Ⅳ3, Ⅳ20) was performed and the candidate gene pool was downsized to 51 genes. Considering that most pathogenic variants affect highly conserved sequences and/or are predicted to be deleterious, we used PolyPhen to assess the flitted non-synonymous variants for a likely functional impact (Table 3). The functional prediction left only 21 variants to be further scanned (S1 Table), of which 7 can be expressed in the nervous system. In further segregation analyses, we used Sanger sequencing to screen for the 7 variants in all clinically affected subjects and eight presumptive unaffected family members in the family, and found that only the N279K missense mutation in *MAPT*, which has previously been reported to cause frontotemporal dementia and parkinsonism linked to chromosome 17 (FTDP-17) [8–12], completely co-segregated with the phenotype in this family (Fig 5).

**Table 3:** Direct identification of the causal gene for the family by exome sequencing.

	Ⅳ3	Ⅳ20	Ⅳ3 and Ⅳ20 shared
Ns/Indel/SV	32492	32417	15215
Non-syn(missense/nonsense/splicesites/readthrough)	6968	6991	3250
Not in dbSNP 129	911	930	178
not in dbSNP129 nor Hapmap8	650	666	83
not in dbSNP129 nor Hapmap8 nor 1000 genomes	484	457	51
Predicted to be deleterious	206	203	21

Rows show the stepwise screening of the total non-synonymous/splice acceptor and donor site/insertions or deletions (NS/SS/Indel) to exclude those found in dbSNP129, the eight HapMap exomes, the dbSNP 1000 genomes, or all. Columns indicate the the number of NS/SS/Indel variants observed in each affected individual (Columns 2–3) or both affected individuals (Columns 4) following exclusion criteria.

## Discussion

Kindreds with FTDP-17 can be divided into the following two major clinical groups based on the phenotypes: dementia predominant and parkinsonism predominant [8]. Families in which dementia is the predominant form are more common, with the clinical features dominated by progressive dementia and personality changes, whereas familial forms with predominant parkinsonism are highly variable. The parkinsonism seen in FTD patients is usually characterized by akinetic-rigid syndrome but in some cases, patients may present symptoms of classical Parkinson's diseases, atypical parkinsonism resembling progressive supranuclear palsy (PSP) or corticobasal syndrome (CBS) [12,13]. The phenotype of the family described in this report was quite different from that of the dementia-predominant kindreds and shares more similarities with the parkinsonism-predominant phenotype at the early stage. Besides, the patients had good initial response to anti-parkinsonian drugs, which prompted an early misdiagnosis as “Parkinson’s disease” having not obtained detailed family history at first. However, as the disease progressed, psychiatric symptoms and personality changes began to appear, accompanied with pyramidal signs and cognitive impairments. All the above changes urged us to have the correct diagnosis of patients with early parkinsonian symptoms.

It has been a long time that PET brain imaging has been applied in detection of brain function under normal and disease conditions [14]. Specifically, glucose metabolism brain imaging using ^18^F-fluorodeoxyglucose (FDG) remains invaluable for differential diagnosis of patients with parkinsonism, who may display distinctive features of cerebral glucose metabolism due to different primary diseases that mimic the presentations of PD. Besides, the dopaminergic-specific radio-ligands have been developed to assist with differential diagnosis, such as ^11^C-CFT [15], ^11^C-raclopride [16], ^11^C-DTBZ [17], ^18^F-dopa [18], which are involved in the presynaptic dopamine activities of dopamine transporter, dopamine receptors, vesicle transporter and dopamine storage, respectively. Although the imaging results could reveal distinctive atrophy in frontotemporal lobe combined with extensive prefrontal area hypometabolism and decreased metabolism in limited premotor and posterior parietal cortical regions, they cannot reach a definitive diagnosis of the disease causes.

According to the International consensus criteria for bvFTD [19], the patients in the pedigree meet the criteria for probable bvFTD, suggesting that we need to further explore the genetic changes in the pedigree to obtain a definitive genetic diagnosis. Thus, we performed exome sequencing for two affected individuals Ⅳ3 and Ⅳ20 and identified heterozygous mutations in the *MAPT* gene as the specific biological basis and the underlying cause of the disease in this family.

*MAPT* gene encodes microtubule-associated protein tau, which is abundant in the axons and stabilizes microtubules and promotes tubulin assembly into microtubules [20]. There are six tau isoforms detected in human brain. Alternative splicing of a 31-amino acid repeats located in the C terminus, which is encoded by exon 10 of the tau gene, results in three tau isoforms with 4 repeats (4R) each and the other three isoforms with 3 repeats (3R) each [21,22]. The 4R tau isoforms stabilize microtubules more strongly than the 3R tau [23]. Mutations in exon 10 of *MAPT* appear to cause disease by disrupting the alternative splicing of exon 10 and hence the ratio of 4R: 3R tau [23]. Abnormal biological processes of tau protein have been associated with a series of neurodegenerative diseases, including AD, PSP, Pick’s disease, and other tauopathies.

So far, 38 mutations of *MAPT* have been reported to be associated with FTDP-17 [24]. The N279K missense mutation is the third most common FTDP-17 mutation, as it was described in five FTDP-17 families from USA, France, Italy and Japan, as well as two unrelated affected individuals from Japan [8–12]. Clinical analysis of previously reported patients suggested that the mutation leads to similar clinical patterns as were observed in this study. The average age at onset ranges from 40 to 50, and the average duration is 6–10 years. Parkinsonism is the first and prominent symptom, whereas at a later phase, symptoms include dementia, pyramidal dysfunction, supranuclear gaze palsy, personality changes, language difficulties or mutism, as well as myoclonus [10, 24].

To our knowledge, our study is the first report to genetically confirm FTDP-17 family in China with parkinsonian symptoms. Unlike previous reports, at least two patients in this family showed good initial response to levodopa for more than one year and three patients manifested typical resting tremor. Moreover, in the ^11^C-CFT DAT and PDRP via ^18^F-FDG PET studies, patients were found to have decreased DAT availability and PD-like metabolic pattern, making the correct diagnosis of the disease quite difficult in the absence of molecular genetic diagnosis. Based on our study, a molecular genetic analysis is essential for the definitive diagnosis of patients with early parkinsonian symptoms.

## Supporting Information

S1 Table The bioinformatics of the 21 overlapping genes after filtration. (DOC)
