# Supplementary material for: Genetic Identification Is Critical for the Diagnosis of Parkinsonism: A Chinese Pedigree with Early Onset of Parkinsonism
Source: PLoS One. 2015 Aug 21;10(8):e0136245. doi: 10.1371/journal.pone.0136245 (PMC4546630; doi:10.1371/journal.pone.0136245)
Supplement: S1 Table — (DOC) [file pone.0136245.s001.doc]

| **Number** | **Gene** | **Chromosome** | **Location** | **sense or antisense strand** | **Base change position** | **Codon change location** | **Exon** | **Codon change** | **Amino acid changes** | **Mutation type** |
| --- | --- | --- | --- | --- | --- | --- | --- | --- | --- | --- |
| 1 | SLIT1 | chr10 | 98796425 | - | mRNA_pos=1829 | codonNum=610 | 20 | CGG>CAG | Arg=>Gln | missense |
| 2 | SEC31B | chr10 | 102245296 | - |  |  | 8 | T>C |  | spliceSite |
| 3 | NECAP1 | chr12 | 8139493 | + | mRNA_pos=706 | codonNum=236 | 7 | 'CCT=>TCT' | 'Pro=>Ser' | missense |
| 4 | KRT14 | chr17 | 36993050 | - | mRNA_pos=1237; | codonNum=413 | 3 | 'GCC=>ACC' | 'Ala=>Thr | missense |
| 5 | ADAM11 | chr17 | 40210609 | + | mRNA_pos=1922 | codonNum=641 | 23 | 'GCG=>GTG' | Ala=>Val' | missense |
| 6 | MAPT | chr17 | 41443527 | + | mRNA_pos=837 | codonNum=279 | 8 | AAT=>AAG | 'Asn=>Lys | missense |
| 7 | GNA13 | chr17 | 60480147 | - | mRNA_pos=445 | codonNum=149; | 3 | ATA=>GTA | Ile=>Val | missense |
| 8 | CCDC46 | chr17 | 61328821 | - | mRNA_pos=2074; | codonNum=692; | 8 | CGG=>TGG'; | Arg=>Trp'; | missense |
| 9 | TMEM54 | chr1 | 33133750 | - | mRNA_pos=452; | codonNum=151; | 3 | CGC=>CAC'; | Arg=>His | missense |
| 10 | RUNX1 | chr21 | 35086349 | - | mRNA_pos=1396 | codonNum=466; | 1 | ATG=>TTG | Met=>Leu | missense |
| 11 | FAM3B | chr21 | 41616737 | + | mRNA_pos=37 | codonNum=13; | 2 | TTC=>GTC' | Phe=>Val' | missense |
| 12 | TMPRSS3 | chr21 | 42676230 | - | mRNA_pos=763 | codonNum=255; | 6 | GCT=>TCT | Ala=>Ser | missense |
| 13 | TMPRSS3 | chr21 | 42682217 | - | mRNA_pos=212; | codonNum=71; | 10 | 'TTC=>TCC | Phe=>Ser | missense |
| 14 | ZNF638 | chr2 | 71430361 | + | mRNA_pos=769; | codonNum=257; | 1 | GTG=>ATG | Val=>Met | missense |
| 15 | NDUFA10 | chr2 | 240609343 | - | mRNA_pos=404; | codonNum=135; | 8 | TTG=>TCG | Leu=>Ser | missense |
| 16 | GPC1 | chr2 | 241053015 | + | mRNA_pos=1084; | codonNum=362; | 6 | GGC=>AGC | Gly=>Ser | missense |
| 17 | GUCA1A | chr6 | 42249453 | + | mRNA_pos=124; | codonNum=42; | 1 | TTC=>ATC | Phe=>Ile | missense |
| 18 | C9orf66 | chr9 | 205228 | - | mRNA_pos=169; | codonNum=57; | 1 | GCC=>CCC | Ala=>Pro | missense |
| 19 | KCNV2 | chr9 | 2708887 | + | mRNA_pos=1148; | codonNum=383; | 1 | CGC=>CAC | Arg=>His | missense |
| 20 | ZXDB | chrX | 57635595 | + | mRNA_pos=389; | codonNum=130; | 1 | GGC=>GAC | Gly=>Asp' | missense |
| 21 | MAGEE2 | chrX | 74920269 | - | mRNA_pos=1343; | codonNum=448; | 1 | CGC=>CAC | Arg=>His | missense |

S1 Table: The [bioinformatics](http://www.baidu.com/link?url=0-XFQXd_pudFwtymOGXVryYX5zI1j4F4cVfcKPth1nucjzjUVW1inqDTAKoaF4R-mj4Y3k4T8Gq_3Nnjy8oTxia_QIus32bVMTWXj4IUU63RCG3S8r56jO9gon2Ddi84) of the 21 overlapping genes after filtration
